# Influence of the Mechanical Environment on the Engineering of Mineralised Tissues Using Human Dental Pulp Stem Cells and Silk Fibroin Scaffolds

**DOI:** 10.1371/journal.pone.0111010

**Published:** 2014-10-29

**Authors:** Anna Woloszyk, Sabrina Holsten Dircksen, Nagihan Bostanci, Ralph Müller, Sandra Hofmann, Thimios A. Mitsiadis

**Affiliations:** 1 Orofacial Development and Regeneration, Institute of Oral Biology, Centre of Dental Medicine, University of Zurich, Zurich, Switzerland; 2 Institute for Biomechanics, ETH Zurich, Zurich, Switzerland; 3 Oral Translational Research, Institute of Oral Biology, Centre of Dental Medicine, University of Zurich, Zurich, Switzerland; 4 Department of Biomedical Engineering, Eindhoven University of Technology, Eindhoven, the Netherlands; 5 Institute for Complex Molecular Sciences, Eindhoven University of Technology, Eindhoven, the Netherlands; Instituto Butantan, Brazil

## Abstract

Teeth constitute a promising source of stem cells that can be used for tissue engineering and regenerative medicine purposes. Bone loss in the craniofacial complex due to pathological conditions and severe injuries could be treated with new materials combined with human dental pulp stem cells (hDPSCs) that have the same embryonic origin as craniofacial bones. Optimising combinations of scaffolds, cells, growth factors and culture conditions still remains a great challenge. In the present study, we evaluate the mineralisation potential of hDPSCs seeded on porous silk fibroin scaffolds in a mechanically dynamic environment provided by spinner flask bioreactors. Cell-seeded scaffolds were cultured in either standard or osteogenic media in both static and dynamic conditions for 47 days. Histological analysis and micro-computed tomography of the samples showed low levels of mineralisation when samples were cultured in static conditions (0.16±0.1 BV/TV%), while their culture in a dynamic environment with osteogenic medium and weekly µCT scans (4.9±1.6 BV/TV%) significantly increased the formation of homogeneously mineralised structures, which was also confirmed by the elevated calcium levels (4.5±1.0 *vs.* 8.8±1.7 mg/mL). Molecular analysis of the samples showed that the expression of tooth correlated genes such as *Dentin Sialophosphoprotein* and *Nestin* were downregulated by a factor of 6.7 and 7.4, respectively, in hDPSCs when cultured in presence of osteogenic medium. This finding indicates that hDPSCs are able to adopt a non-dental identity by changing the culture conditions only. Also an increased expression of *Osteocalcin* (1.4x) and *Collagen type I* (1.7x) was found after culture under mechanically dynamic conditions in control medium. In conclusion, the combination of hDPSCs and silk scaffolds cultured under mechanical loading in spinner flask bioreactors could offer a novel and promising approach for bone tissue engineering where appropriate and rapid bone regeneration in mechanically loaded tissues is required.

## Introduction

Hard tissues of the craniofacial complex are subjected to physical, chemical and biological health risk factors of the surrounding environment. Examples are high mechanical impact resulting in fractures, smoking as a risk factor for osteoporosis and bone fracture [1], as well as microbiological infections of the periodontal tissues resulting in alveolar bone and subsequent tooth loss [2]. Repair of these bones requires the reconstruction of their anatomical, physiological, and functional properties, which cannot yet be fully accomplished by current treatment strategies [3]. For this reason, a considerable effort has been made during the last two decades to generate innovative engineering products that could be used together with specific populations of stem/progenitor cells and growth factors in order to form the most adequate and physiological substitutes for bone repair.

Mesenchymal cells forming craniofacial bones and dental tissues (e.g. dentin) have the same embryonic origin and share many biochemical and molecular properties [4]–[6]. These tissues are composed mostly of collagen type I and, to a lesser degree, of other tissue-specific extracellular matrix components that with the progressive deposition of hydroxyapatite crystals become highly mineralised [7], [8]. As for all other tissues, their homeostasis is based on the presence of sufficient vasculature [9], innervation [10], and specific stem cell populations. These cells are characterised by their ability to self-renew without losing their potency to differentiate into various cell types [11].

Bones undergo constant remodelling by a well-orchestrated interplay of stem cell-derived osteoblasts and osteoclasts [12]. In contrast to that, dentin is not remodelled but continuously deposited by odontoblasts throughout the life of a tooth [13], [14]. Following serious dental injuries or carious lesions, disintegrated odontoblasts can be replaced by newly-formed odontoblasts that originate from stem cells residing in the dental pulp [5], [14]–[16].

Bone marrow stem cells (BMSCs) were the first to be used in clinics with success for the treatment of leukemias since the 1950 s [17] and thus constitute the golden standard in stem cell research. Since the discovery of dental pulp stem cells (DPSCs) comparative studies have shown that both BMSCs and DPSCs have almost identical properties in terms of gene expression and differentiation potential (e.g. osteogenic, chondrogenic, adipogenic, myogenic, neurogenic) [15], [18], [19]. However, DPSCs exhibit higher clonogenic and proliferative potential when compared to BMSCs [20]. In contrast to BMSCs that originate from the mesoderm, DPSCs are derived from cranial neural crest (CNC) cells, as demonstrated by the expression of markers characteristic for CNC-derived stem cells like GFAP, HNK-1, nestin, P75, and S-100 [11], [21]. Common embryonic origin together with the Hox gene expression profile was shown to play a key role in determining progenitor cell fate during adult bone regeneration [22]. It was demonstrated that Hox-negative neural crest-derived skeletal stem cells adopted a Hox-positive profile and differentiated into osteoblasts after transplantation into a defect in the mesoderm-derived tibia. On the contrary, Hox-positive mesoderm-derived skeletal stem cells did not adopt a Hox-negative profile and differentiated into chondroblasts after transplantation into a defect in the neural crest-derived mandible [22]. This proves that stem cells differ in plasticity and that they have a ‘positional memory’ that depends on their Hox gene expression profile [23]. The use of DPSCs is thus important for reconstitution of craniofacial tissues and presumably much more appropriate than the use of BMSCs. Furthermore, DPSCs are easily accessible after routine tooth extraction procedures resulting in little morbidity and therefore a realistic autologous cell source, while BMSC isolation requires invasive and painful surgery. Optimising the combination of DPSCs, scaffolds, growth factors, and the use of mechanical loading for the generation of particular hard tissues is currently a great challenge.

Numerous types of silk fibroin scaffolds have been used for tissue engineering purposes in the last two decades [24], [25]. Important advantages of silk compared to other biomaterials are the excellent biocompatible and mechanical properties, load-bearing capacity, and lack of releasing toxic by-products during silk degradation [26]. Silk scaffolds have been used in combination with a variety of cell types to obtain the most desirable effects for specific tissue repair. The behaviour and fate of stem cells closely depend on the geometry and composition of the scaffolds, as well as the applied forces [27]. For example, BMSCs were incorporated in hexafluoro-2-propanol based porous silk scaffolds for optimal bone regeneration [28]–[31]. Mechanical cues, including shear stress, substrate stiffness and nano-topography, have been shown to also stimulate osteogenesis [32]. Mechanotransduction is carried out through several mechanoreceptors, like integrins, cadherins, gap junctions, and Ca^2+^ channels, which are able to alter gene expression in response to loading or stretching [33]–[35]. A tool for imposing shear stress on cells during *in vitro* culture conditions is the spinner flask bioreactor through increased turbulent fluid flow, generated by stirring of the medium. Compared to static cultures, the bioreactor improves the culture conditions since it allows better control in terms of temperature, pH, pressure, nutrient and oxygen supply as well as waste removal [36], [37].

After investigating the osteogenic potential of human DPSCs seeded in silk scaffolds cultured in both static and dynamic conditions, in the present study we were able to show that these cells are reactive to mechanical loading, which is an important component of both the bone and the tooth environment and is able to increase the mineralisation of silk scaffolds by human DPSCs *in vitro*.

## Materials and Methods

### Preparation of silk fibroin scaffolds

Silk fibroin scaffolds were prepared as previously described [29]. Briefly, silkworm cocoons (Trudel Inc., Zurich, Switzerland) were boiled 2 times for 1 h in an aqueous solution of 0.02 M Na_2_CO_3_ (Fluka, Buchs SG, Switzerland) and rinsed with ultrapure water (UPW) to extract sericin. Lyophilized silk was dissolved in hexafluoro-2-propanol (HFIP) (abcr GmbH & Co., Karlsruhe, Germany) to obtain a 17% (w/v) silk fibroin solution. NaCl crystals (224–315 µm in diameter) (Sigma-Aldrich, Buchs SG, Switzerland) were weighed in Teflon containers and silk fibroin/HFIP solution was added at a ratio of 20: 1 (NaCl/silk fibroin). After complete evaporation of the solvent NaCl/silk fibroin blocks were immersed in 90% (v/v) methanol (Sigma-Aldrich, Buchs SG, Switzerland) in UPW for 30 min, dried and NaCl was leached by incubation in UPW for 2 days resulting in scaffolds with more than 90% porosity [38]. 25 disk shaped scaffolds were punched (10 mm diameter, 1–2 mm thick) and autoclaved at 121°C and 1 bar for 20 min.

### Isolation, culture and characterisation of dental pulp stem cells (DPSCs)

DPSCs were obtained from dental pulps of clinically healthy teeth from 14–16 year old patients who had their teeth extracted due to orthodontic treatment. The procedure was reviewed and approved by the Institutional Review Board of the Centre of Dental Medicine, University of Zurich and was performed with guardians’ and underage patients’ written informed consent abiding by the guidelines for studies with human cells of irreversibly anonymous origin. Written consents are stored in the dental faculty for all anonymized studies. After removal of the dental pulp from the tooth, DPSCs were isolated, expanded and characterized as described earlier [39]. All experiments were performed with DPSCs at passage 3 (P3) or P4.

### Scaffold seeding

25 silk fibroin scaffolds were pre-wetted in Dulbecco's Modified Eagle Medium (DMEM) for 36 hours at room temperature before seeding them with DPSCs (5 scaffolds per group) at a density of 5×10^6^ cells per scaffold in 50 µL of control medium (DMEM, 10% FBS, 1% Pen-Strep and 50 ng/mL fungizone). To allow attachment of DPSCs to the scaffolds, they were incubated for 90 min in a humidified incubator at 37°C. Thereafter, 1 mL culture medium per well (12-well-plate) was added and scaffolds were incubated for additional 24 h. Subsequently, scaffolds were placed in spinner flask bioreactors with a stirring speed of 120 rpm (referred to as dynamic) or in spinner flask bioreactors without stirring (referred to as static) and were cultured in either control or osteogenic medium (control medium plus 50 µg/mL ascorbic acid-2-phosphate, 100 nM dexamethasone, 7 mM β-glycerolphosphate) at 37°C with 5% CO_2_. All cell culture ingredients were obtained from Gibco Invitrogen, Basel, Switzerland.

### Sample analysis

#### Micro-computed tomography (µCT)

µCT measurements were performed on a µCT80 imaging system (Scanco Medical, Brüttisellen, Switzerland). Samples were scanned either weekly starting from day 19 (groups Sp.O.W, Sp.C.W) or on the last day of the experiment only to determine potential x-ray effects on cell development (groups Sp.O.E, St.O.E, St.C.E) (*n* = 5–6 per group). Scanning was performed at an isotropic nominal resolution of 18 µm, energy level was set to 45 kVp, intensity to 177 µA, 200 ms integration time and two-fold frame averaging. A constrained Gaussian filter was applied to reduce part of the noise. Filter support was set to 1.0 and filter width sigma to 0.8. Segmentation was performed to distinguish mineralised tissue from non-mineralised tissue. A threshold that corresponded to a hydroxyapatite density of 145 mg HA/ccm was set after visual judgement of the grey images to identify mineralised structures. Components smaller than 50 voxels were filtered away by applying component labelling. Quantitative morphometry was performed to assess relative mineralised extracellular matrix volume of the entire construct using direct microstructural bone analysis as previously described for human bone biopsies [40].

After 47 days of culture, scaffolds were blotted on clean paper towels to determine the total wet weight. Scaffolds were weighed, cut into six pieces, weighed again and processed for various analyses (*n* = 5–6 per group). Where necessary, samples were disintegrated using steel balls and a Mini-Beadbeater (Biospec, Bartlesville, OK, USA) three times at 25,000 rpm for 10 s with cooling of the samples in between the cycles.

#### Cell proliferation and viability

Cell proliferation was determined by DNA analysis from disintegrated samples. DNA content was measured using the Quant-iT PicoGreen dsDNA Assay Kit (Life Technologies Europe B.V., Zug, Switzerland) and cell viability was evaluated using the AlamarBlue assay (Life Technologies Europe B.V., Zug, Switzerland) according to the manufacturer's instructions. Fluorescence was measured using a plate reader (Infinite 200 PRO, Tecan Group Ltd, Männedorf, Switzerland).

#### Osteogenic potential of DPSCs

To study the osteogenic capacity of DPSCs seeded into silk fibroin scaffolds we performed alkaline phosphatase (ALP) and calcium deposition assays additionally to the µCT evaluation. For this purpose, disintegrated scaffold pieces were used. ALP activity (Sigma-Aldrich, Buchs SG, Switzerland) was measured spectrophotometrically based on conversion of p-nitrophenyl phosphate to p-nitrophenol as previously described [29]. Quantification of calcium was performed with the Calcium (CPC) LiquiColor Test according to the manufacturer's instructions (DiaSys Greiner, Flacht, Germany). Collagen type I was assessed by staining the intact samples with Sirius Red (Sigma-Aldrich, Buchs SG, Switzerland) as described previously [41]. After washing the samples with UPW, the remaining colour in saturated picric acid was bleached in a 1: 1 mix of Methanol and 0.2 M NaOH (Sigma-Aldrich, Buchs SG, Switzerland).

#### Histology

After fixation of the constructs in 10% neutral buffered formalin at room temperature for 20 min and 5 times washing with UPW, samples were dehydrated and impregnated with paraffin over night by use of a tissue processor (TPC 15 Duo, Medite AG, Winter Garden FL, United States) before being embedded in paraffin using a paraffin embedding station (TES 99, Medite AG, Winter Garden, FL, USA). Paraffin sections of 6–7 µm (HM355S, Microm International, Walldorf, Germany) were stained with Haematoxylin and Eosin (H&E) (Sigma-Aldrich, Buchs SG, Switzerland) for a general overview. Collagen distribution was visualised by Sirius Red staining while mineralisation was shown with von Kossa staining (Sigma-Aldrich, Buchs SG, Switzerland).

#### Gene expression analysis

RNA was extracted using a RNA extraction kit (RNeasy Mini Kit, Qiagen, Basel, Switzerland) and then transcribed into cDNA (High Capacity cDNA Reverse Transcription Kit, Applied Biosciences, Foster City, CA, USA). Quantitative Real Time (qRT) PCR was performed using SYBR-Green-based protocols in a StepOne Real Time PCR System (Applied Biosystems, Life Technologies, Basel, Switzerland). Expression analysis of *ALP*, *Collagen type I*, *Dentin Sialophosphoprotein (DSPP)*, *Nestin*, *Osteocalcin* and *GAPDH* (housekeeping gene) were carried out using the qPCR SYBR Master Mix (Applied Biosystems, Carlsbad, USA) in combination with oligonucleotide primers (Tab. 1), specifically designed for the indicated genes. Expression levels were calculated by the comparative ΔCt method (2^−ΔCt^ formula) after being normalised to the Ct-value of the *GAPDH* housekeeping gene.

**Table 1:** Primers used in the study.

Primers	Sequence	Function
*Alkaline Phosphatase (ALP)*	fw ATGAAGGAAAAGCCAAGCAG	marker for matrix mineralisation
	rv ATGGAGACATTCTCTCGTTC	
*Collagen type I (Col1)*	fw AAGATGGACTCAACGGTCTC	marker for bone formation
	rv CAGGAAGCTGAAGTCGAAAC	
*Dentin sialophosphoprotein*	fw GAATTCTGCTGGTATTCCAG	marker for dentinogenesis
*(DSPP)*	rv GCCATTAGATTCATCACTGC	
*Glyceraldehyde 3-phosphate*	fw ATCACTGCCACCCAGAAGAC	housekeeping gene
*dehydrogenase (GAPDH)*	rv ATGAGGTCCACCACCCTGTT	
*Nestin*	fw CCTGCAAAAGGAGAATCAAG	marker for cell proliferation/migration,
	rv GTTCTCAATGTCTCTTGGTC	marker for odontoblasts
*Osteocalcin (OC)*	fw TCTCTGCTCACTCTGCTGG	marker for osteoblasts
	rv GCGTTTGTAGGCGGTCTTC	

### Statistical analysis

Comparisons of the groups were performed using one-way analysis of variance (ANOVA). When there were significant differences (*p*<0.05), comparisons betweeen the groups were further assessed with Bonferroni multiple-comparison test. Data were considered statistically significant at *p*<0.05 and highly significant at *p*<0.01.

## Results

### Mineralised tissue formation by hDPSCs

**Figure 1:**
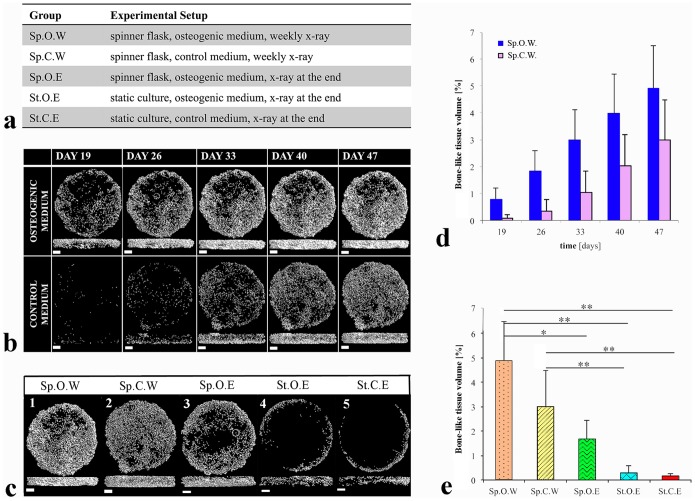
Mineralisation analysis using µCT. (**a**) Experimental groups. (**b**) Time-lapse µCT images of samples cultured in spinner flasks in either osteogenic (upper row) or control (lower row) medium. (**c**) µCT images of one representative sample per experimental group after 47 days of culture. (**d**) Time-dependent increase in bone-like tissue volume fraction as observed with weekly µCT scans. (**e**) Bone-like tissue volume fraction for all groups after 47 days of culture. Scale bars: 1 mm.

Mineralisation was monitored by time-lapse µCT starting from day 19 of *in vitro* culture in two of the five experimental groups, which were both cultured in a mechanically dynamic system in either osteogenic (Sp.O.W) or control medium (Sp.C.W) (Fig. 1b). Cells grown in control medium formed mineralised matrix with a delay of 2 weeks (Fig. 1d). A comparison of all the groups (Fig. 1a) showed significantly higher mineralisation in samples cultured at 120 rpm (dynamic) than samples cultured at 0 rpm (static) (*p*<0.01) (Fig. 1c, 1e), being even more abundant in samples that were cultured additionally in osteogenic medium (Fig. 1b, 1d). Mineralisation followed a linear pattern (Fig. 1d) with correlation coefficients of 0.99 and 0.98 for samples cultured in osteogenic and control medium, respectively. Both qualitative and quantitative analyses of the bone-like tissue volume fraction for all groups demonstrated that mineralisation was significantly higher in DPSC-seeded scaffolds cultured in spinner flasks at 120 rpm in osteogenic medium with weekly µCT scans (Fig. 1c1, 1e) when compared to the static control samples (Fig. 1c4, 1c5, 1e) (*p*<0.01). Samples cultured at 120 rpm (Fig. 1c1–3) exhibited a more homogenous mineralisation than samples cultured at 0 rpm (Fig. 1c4, 1c5).

### Histological analysis of the tissue formed by DPSCs

**Figure 2:**
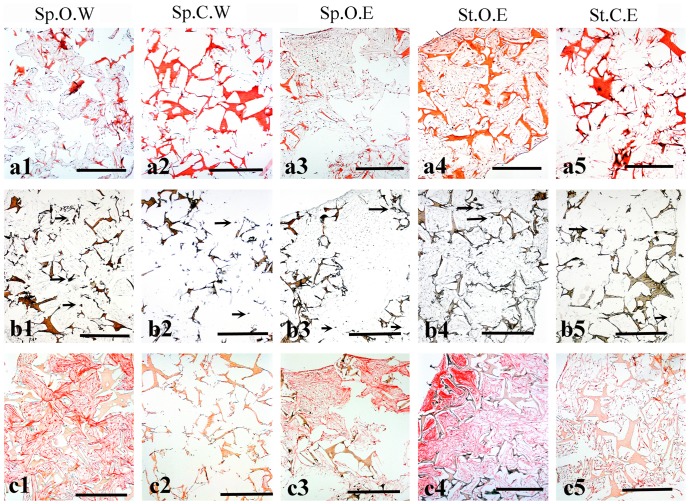
Histological analysis of hDPSCs seeded on silk fibroin scaffolds after 47 days of culture. (**a**) Haematoxylin and Eosin staining of histological sections from all groups. Extracellular matrix and silk scaffolds are stained red/pink. Cell nuclei are stained purple/violet. (**b**) Von Kossa staining. Silk scaffolds and mineralised nodules are stained in brown. Arrows indicate areas where phosphate is present. (**c**) Sirius Red staining showing the distribution of Collagen (red colour). Abbreviations: Sp.O.W: spinner flask culture in osteogenic medium with weekly x-ray; Sp.C.W: spinner flask culture in control medium with weekly x-ray; Sp.O.E: spinner flask culture in osteogenic medium with x-ray at the end; St.O.E: static culture in osteogenic medium with x-ray at the end; St.C.E: static culture in control medium with x-ray at the end. Scale bars: 500 µm.

Haematoxylin and Eosin staining showed that in samples cultured in spinner flasks the cells were distributed all over the scaffolds (Fig. 2a1–2a3). In their centre these scaffolds presented larger hollow areas free of cells and extracellular matrix (ECM). The amount of cells and ECM appeared to be lower in samples cultured in control medium (Fig. 2a2, 2a5) when compared to cells grown in osteogenic medium (Fig. 2a1, 2a4). The von Kossa staining confirmed the presence of phosphate in all groups (Fig. 2b1–2b5) and the Sirius Red staining allowed the visualisation of collagen type I fibres in all groups (Fig. 2c1–2c5). Qualitatively, the most homogenous distribution of collagen fibres was found in samples cultured in osteogenic medium in spinner flasks with weekly µCT scans (Fig. 2c1). Both controls showed a very weak signal due to the low amount of collagen produced by the cells (Fig. 2c2, 2c5), which was confirmed by the Sirius Red assay (results not shown) where average collagen concentrations were highest in groups that had been cultured in osteogenic medium. Collagen concentrations in both static and dynamic samples with µCT at the end of the culture (Sp.O.E, St.O.E) were shown to be significantly higher than the dynamic sample cultured in control medium with weekly µCT scans (Sp.C.W) with *p*<0.05 and *p*<0.01, respectively.

### Biochemical analysis

**Figure 3:**
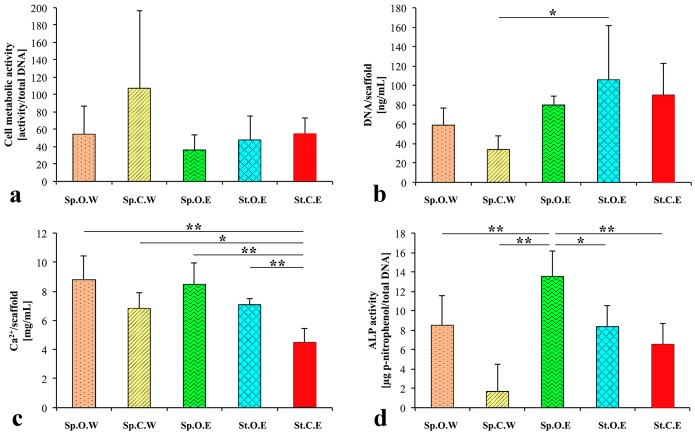
Biochemical analysis after 47 days of culture. (**a**) Cell metabolic activity [activity/total DNA]. (**b**) DNA content per scaffold [ng/mL]. (**c**) Calcium content per scaffold [mg/mL]. (**d**) ALP activity normalised to cell number [µg/p-nitrophenol/total DNA]. Data is shown as average ± standard deviation (*n* = 5). Asterisks indicate significant (*p*<0.05) or highly significant (*p*<0.01) difference between the groups. Abbreviations: Sp.O.W: spinner flask culture in osteogenic medium with weekly x-ray; Sp.C.W: spinner flask culture in control medium with weekly x-ray; Sp.O.E: spinner flask culture in osteogenic medium with x-ray at the end; St.O.E: static culture in osteogenic medium with x-ray at the end; St.C.E: static culture in control medium with x-ray at the end.

The AlamarBlue assay showed that cell viability was not significantly different between the groups. However, cells cultured in the control medium were slightly more active than cells cultured in osteogenic medium (e.g. Sp.C.W = 107.2±89.8 *vs*. Sp.O.W = 54.3±32.5) (Fig. 3a). Furthermore, the number of cells was not significantly different between the groups except between samples cultured at 120 rpm in control medium with weekly µCT scans (Sp.C.W) and samples cultured at 0 rpm in osteogenic medium with one µCT scan in the end of the study (St.O.E) (*p*<0.05) (Fig. 3b). Calcium deposition was significantly increased 1.5–2 fold (*p*<0.05) when cells were cultured in osteogenic medium and/or in spinner flasks at 120 rpm (Fig. 3c). ALP activity, which is a marker for odontoblastic and osteoblastic differentiation, was negatively influenced by weekly µCT scans (*p*<0.01) as well as by static culture conditions (*p*<0.05). The highest level of ALP activity (13.5±2.7 µg p-nitrophenol/total DNA) was measured in samples cultured at 120 rpm with osteogenic medium and without weekly µCT scans (Fig. 3d).

### Gene expression analysis

**Figure 4:**
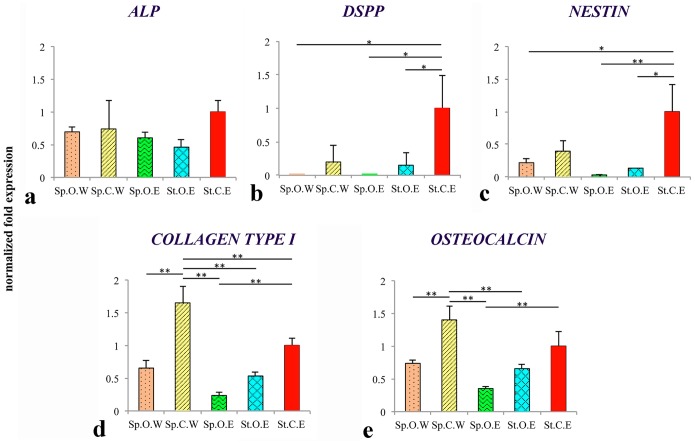
Gene expression analysis. Images showing normalised fold expression against *GAPDH* for the genes *ALP* (**a**), *DSPP* (**b**), *Nestin* (**c**), *Collagen type I* (**d**) and *Osteocalcin* (**e**). Data is shown as average ± standard deviation of three samples. Asterisks indicate significant (*p*<0.05) or highly significant (*p*<0.01) difference between the groups. Abbreviations: Sp.O.W: spinner flask culture in osteogenic medium with weekly x-ray; Sp.C.W: spinner flask culture in control medium with weekly x-ray; Sp.O.E: spinner flask culture in osteogenic medium with x-ray at the end; St.O.E: static culture in osteogenic medium with x-ray at the end; St.C.E: static culture in control medium with x-ray at the end.

*ALP*, *DSPP* and *Nestin* expressions were downregulated in the experimental groups after 47 days of culture when compared to the control groups (Fig. 4a–c). *ALP* downregulation by a factor of 1.4–2.2 was not significant, whereas in the case of *DSPP* and *Nestin* the gene expression was significantly downregulated (*p*<0.05) in all groups except in the samples cultured at 120 rpm with control medium and weekly µCT scans (Sp.C.W). Similar expression patterns were observed between *Collagen type I* and *Osteocalcin* (Fig. 4d, e). In samples cultured dynamically (at 120 rpm) with control medium and weekly µCT scans (Sp.C.W) gene expression was higher by 1.6 fold and 1.4 fold (*p*<0.01), respectively, when compared to the control, with a highly significant increase in the case of *Collagen type I*. All remaining experimental groups were downregulated by a factor of 1.4–4.2, with only the samples cultured at 120 rpm in osteogenic medium with a µCT scan at the end of the experiment (Sp.O.E) being significantly different (*p*<0.01) for both *Collagen type I* and *Osteocalcin*.

## Discussion

The dental pulp is an easily accessible source of multipotent cell populations. Therefore, the goal of this study was to investigate the potential of dental pulp stem cells (DPSCs) to be used for the regeneration of bone tissue and if mechanical loading could improve the behaviour of this cell population. For this purpose, human DPSCs (hDPSCs) were seeded on silk fibroin scaffolds that offer a three-dimensional (3D) environment, which has been shown to allow proper cell adhesion and proliferation [42] and were cultured in static (0 rpm) and dynamic (120 rpm) conditions.

Mechanical loading has previously been shown to positively affect proliferation, differentiation, and ECM production when tension was applied on hDPSCs in two-dimensional (2D) and 3D cultures [43]. In line with the previous findings, the present study demonstrates that the application of mechanical loading in the form of turbulent flow accelerates the process of mineral deposition. Additionally, biochemical analyses have confirmed that the spinner flask culture conditions promote the differentiation capability of hDPSCs into mineral producing cells while they reduce their proliferation activity. This is not surprising since recent studies have shown that proliferation and differentiation cannot progress at the same time [44]. Indeed, hDPSCs that were exposed to uniaxial mechanical stretch increased their proliferation rate, while their differentiation into osteoblasts was dramatically decreased [45]. In contrast, hDPSCs stimulated by hydrostatic pressure increased their differentiation rate despite their reduced number and adhesion capacity [46]. In the present study we showed that the application of mechanical loading in the form of flow accelerated the process of mineral deposition. Biochemical analyses indicated higher cell proliferation activity in the statically cultured samples, while under dynamic culture conditions the differentiation capability of hDPSCs into mineral producing cells was promoted. This result was also confirmed by the amount of mineralised ECM deposition as evaluated by µCT. Statically (0 rpm) cultured scaffolds were mineralised only on their top and edges, whereas a stirring speed of 120 rpm resulted in a more homogeneous mineralisation in the scaffolds. These low levels of mineralisation in statically cultured samples could be explained by the limited nutrient supply in 3D scaffolds that exceed the size of 1 mm, as pointed out by previous *in* *vitro* studies where the formation of tissues was problematic in the centre of the scaffold [47], [48]. By applying mechanical loading the nutrient supply could be improved.

Although there have been some attempts to regenerate lost alveolar bone (specialised bone structure that supports tooth) in patients using autologous hDPSCs seeded onto collagen sponge scaffolds [49], the results are still unsatisfactory. In these studies, hDPSCs have been isolated from third molars and seeded onto collagen sponge scaffolds before their implantation at the defective jaw sites. X-rays and histological analyses have shown that new bone was formed at the implantation sites three months post-surgery. Follow-up studies (three years post-surgery) have revealed that the regenerated bone was more compact than the physiological one [49]. These *in vivo* results clearly show that hDPSCs are able to differentiate into osteoblasts and repair bone defects in the orofacial area in a mechanically loaded environment. However, the density of the bone produced by hDPSCs when seeded onto collagen scaffolds was more compact than alveolar bone, which could compromise its metabolic functions due to the decrease in porosity. High bone density could be the result of the fast degradation time (4–5 weeks) of the collagen sponge [50], thus resulting in the lack of mechanical support before bone formation is completed.

Silk fibroin scaffolds could be an alternative to collagen materials since they perform better than collagen scaffolds sharing similar microstructures [51]–[53]. Silk fibroin is a biocompatible (after the removal of sericin) and biodegradable material whose physical and mechanical properties can be easily manipulated through structural readjustments [25], [26], [54]. Compared to collagen, silk fibroin offers a higher mechanical stability and a much slower degradation rate [25]. It has been demonstrated that bone-like tissue deposits occur appositionally to the silk fibroin scaffold, suggesting that the internal geometry of the scaffold might be used to determine the structure of the engineered bone [29], [51]. At the same time this effect could not be accomplished with collagen scaffolds due to their faster degradation and the resulting loss of mechanical stability and geometrical guidance for the incorporated cells. Histological analyses confirmed high cell compatibility of silk fibroin scaffolds under dynamic conditions with a good amount of ECM being produced by hDPSCs over most of the scaffold volume.

Real Time PCR studies showed that hDPSCs cultured in osteogenic medium lost their dental genotypic profile, as indicated by the decreased levels of *DSPP* and *Nestin* expression when compared to the control. Even though *DSPP* has been also found to be expressed in bone, cementum, and certain non-mineralised tissues, the expression levels in these tissues were shown to be much lower than in dentin [55]. In contrast, expression of *Osteocalcin* that is mainly a bone-specific gene [56] and *Collagen type I*, which is expressed in both bone and dentin tissues, was upregulated in mechanically loaded samples. This has been already demonstrated in several previous studies and has been connected to the improved transport of nutrients [57]–[59].

Interestingly, a significantly positive effect of radiation on the mineralisation of the scaffold was observed during the µCT scans that contradicts previously reported results, where both hard tissue formation and cell survival were significantly reduced [60]–[62]. In a previous study where BMSCs were cultured on silk fibroin scaffolds µCT imaging had no impact on the osteogenic performance of the cells when compared to non-exposed samples [63].

## Conclusion

In summary, by biochemical, histological, and µCT evaluations together with molecular analyses we have shown that hDPSCs have the potential to form mineralised matrix when grown on porous 3D silk fibroin scaffolds. This potential can be enhanced by mechanical loading and the addition of osteogenic factors in the culture medium. The clinical relevance lies in the availability of hDPSCs, their common embryonic origin with craniofacial tissues, and the advantageous characteristics of the silk fibroin scaffold for applications in bone regeneration.

## Supporting Information

File S1 **Tables S1–S3.** Table S1. Dataset of raw values for figure 1d. Table S2. Dataset of raw values for figure 1e and figures 3a–3d. Table S3. Dataset of raw values for figures 4a–4e.
(DOCX)
