# Supplementary material for: Influence of the Mechanical Environment on the Engineering of Mineralised Tissues Using Human Dental Pulp Stem Cells and Silk Fibroin Scaffolds
Source: PLoS One. 2014 Oct 29;9(10):e111010. doi: 10.1371/journal.pone.0111010 (PMC4213001; doi:10.1371/journal.pone.0111010)
Supplement: File S1 — Tables S1–S3. Table S1. Dataset of raw values for figure 1d. Table S2. Dataset of raw values for figure 1e and figures 3a–3d. Table S3. Dataset of raw values for figures 4a–4e. (DOCX) [file pone.0111010.s001.docx]

**Influence of the mechanical environment on the engineering of mineralised tissues using human dental pulp stem cells and silk fibroin scaffolds**

Minimal Dataset

**Table S1.** Dataset of raw values for figure 1d.

| **Sample** | **Fig. 1d** | |
| --- | --- | --- |
|  | Sp.O.W | Sp.O.C |
| 19 days_s1 | 0.784746 | 0.312726 |
| 19 days_s2 | 0.259674 | 0.006528 |
| 19 days_s3 | 0.476005 | 0.024552 |
| 19 days_s4 | 1.074567 | 0.024976 |
| 19 days_s5 | 1.280085 | 0.012269 |
| **19 days_AVG** | **0.775015** | **0.07621** |
| **19 days_STD** | **0.418302** | **0.132455** |
| 26 days_s1 | 1.98261 | 1.065473 |
| 26 days_s2 | 0.867818 | 0.030651 |
| 26 days_s3 | 1.222251 | 0.362495 |
| 26 days_s4 | 2.381141 | 0.222686 |
| 26 days_s5 | 2.691861 | 0.103335 |
| **26 days_AVG** | **1.829136** | **0.356928** |
| **26 days_STD** | **0.768928** | **0.415588** |
| 33 days_s1 | 3.482533 | 2.1763 |
| 33 days_s2 | 1.672943 | 0.181636 |
| 33 days_s3 | 1.943115 | 1.435473 |
| 33 days_s4 | 3.469906 | 0.838599 |
| 33 days_s5 | 4.319967 | 0.586203 |
| **33 days_AVG** | **2.977693** | **1.043642** |
| **33 days_STD** | **1.126007** | **0.77955** |
| 40 days_s1 | 4.853936 | 3.450746 |
| 40 days_s2 | 2.456252 | 0.50974 |
| 40 days_s3 | 2.504989 | 2.856389 |
| 40 days_s4 | 4.26687 | 1.876243 |
| 40 days_s5 | 5.788802 | 1.413683 |
| **40 days_AVG** | **3.97417** | **2.02136** |
| **40 days_STD** | **1.467576** | **1.163587** |
| 47 days_s1 | 6.06193 | 4.638431 |
| 47 days_s2 | 3.369953 | 0.928202 |
| 47 days_s3 | 3.113331 | 4.168518 |
| 47 days_s4 | 5.239346 | 2.934587 |
| 47 days_s5 | 6.670735 | 2.358429 |
| **47 days_AVG** | **4.891059** | **3.005633** |
| **47 days_STD** | **1.591664** | **1.479693** |

**Table S2.** Dataset of raw values for figure 1e and figures 3a-3d.

| **Sample** | **Fig. 1e** | **Fig. 3a** | **Fig. 3b** | **Fig. 3c** | **Fig. 3d** |
| --- | --- | --- | --- | --- | --- |
| Sp.O.W_s1 | 6.06193 | / | 57.06955 | 6.035828 | 7.732995 |
| Sp.O.W_s2 | 3.369953 | / | 78.13824 | 8.90254 | 5.720643 |
| Sp.O.W_s3 | 3.113331 | / | 70.62268 | 8.751287 | 5.706586 |
| Sp.O.W_s4 | 5.239346 | 31.29321 | 58.06371 | 9.787815 | 12.55618 |
| Sp.O.W_s5 | 6.670735 | 77.23739 | 30.64578 | 10.41837 | 10.7347 |
| **Sp.O.W_AVG** | **4.891059** | **54.2653** | **58.90799** | **8.779167** | **8.490221** |
| **Sp.O.W_STD** | **1.591664** | **32.48744** | **18.09614** | **1.676771** | **3.064404** |
| Sp.C.W_s1 | 4.638431 | 62.06018 | 40.1868 | 7.874809 | 3.324021 |
| Sp.C.W_s2 | 0.928202 | 48.2903 | 44.37744 | 6.169112 | -1.07401 |
| Sp.C.W_s3 | 4.168518 | / | / | 6.408459 | -1.60438 |
| Sp.C.W_s4 | 2.934587 | 77.69622 | 38.08422 | 8.040718 | 3.299962 |
| Sp.C.W_s5 | 2.358429 | 240.5967 | 12.40665 | 5.611756 | 4.425877 |
| **Sp.C.W_AVG** | **3.005633** | **107.1609** | **33.76378** | **6.820971** | **1.674293** |
| **Sp.C.W_STD** | **1.479693** | **89.76471** | **14.4764** | **1.078847** | **2.794572** |
| Sp.O.E_s1 | 2.488279 | 20.70992 | 92.85404 | 8.344058 | 9.829305 |
| Sp.O.E_s2 | 1.153119 | 49.97207 | 68.17808 | 8.335852 | 13.69327 |
| Sp.O.E_s3 | 1.354779 | 27.63219 | 85.00956 | 10.87167 | 12.31398 |
| Sp.O.E_s4 | 2.476143 | 22.5688 | 75.98987 | 8.073511 | 16.81943 |
| Sp.O.E_s5 | 0.928119 | 59.64982 | 76.19469 | 6.733018 | 14.98217 |
| **Sp.O.E_AVG** | **1.680088** | **36.10656** | **79.64525** | **8.471622** | **13.52763** |
| **Sp.O.E_STD** | **0.747639** | **17.59744** | **9.486995** | **1.498012** | **2.651868** |
| St.O.E_s1 | 0.301424 | 51.84457 | 77.52017 | 7.108653 | 7.166136 |
| St.O.E_s2 | 0.794752 | 25.15717 | 93.41274 | 7.58421 | 9.218532 |
| St.O.E_s3 | 0.18004 | 77.35009 | 69.41168 | 6.609426 | 11.35579 |
| St.O.E_s4 | 0.416545 | 69.63865 | 82.69833 | 7.529188 | 8.504102 |
| St.O.E_s5 | 0.039232 | / | / | 6.711591 | / |
| St.O.E_s6 | 0.021012 | 14.31663 | 205.2857 | 6.908165 | 5.517771 |
| **St.O.E_AVG** | **0.292168** | **47.66142** | **105.6657** | **7.075205** | **8.352466** |
| **Sp.O.E_STD** | **0.289213** | **27.38718** | **56.36557** | **0.4107** | **2.192936** |
| St.C.E_s1 | 0.031223 | 42.24134 | 107.9038 | 3.862522 | 10.49488 |
| St.C.E_s2 | 0.086777 | 35.83366 | 101.134 | 4.339593 | 7.108021 |
| St.C.E_s3 | 0.283655 | 77.76389 | 70.68833 | 3.639884 | 5.171111 |
| St.C.E_s4 | 0.279137 | 37.56019 | 140.6809 | 4.417854 | 4.726854 |
| St.C.E_s5 | 0.212121 | 73.37046 | 57.69352 | 6.396479 | 6.739669 |
| St.C.E_s6 | 0.096477 | 61.60759 | 59.29464 | 4.221679 | 4.916411 |
| **St.C.E_AVG** | **0.164898** | **54.72953** | **89.56584** | **4.479669** | **6.526158** |
| **St.C.E_STD** | **0.107738** | **18.6181** | **32.7762** | **0.984913** | **2.181736** |

**Table S3.** Dataset of raw values for figures 4a-4e.

| **Sample** | **Fig. 4a** | **Fig. 4b** | **Fig. 4c** | **Fig. 4d** | **Fig. 4e** |
| --- | --- | --- | --- | --- | --- |
| Sp.O.W_s1 | 0.67988534 | 0.01453432 | 0.18466998 | 0.8080589 | 0.80917779 |
| Sp.O.W_s2 | 0.79007997 | 0.00126913 | 0.312739 | 0.63262355 | 0.69859248 |
| Sp.O.W_s3 | 0.612441 | 0.00100316 | 0.15602959 | 0.51859247 | 0.70126861 |
| **Sp.O.W_AVG** | **0.69413544** | **0.00560221** | **0.21781286** | **0.65309164** | **0.73634629** |
| **Sp.O.W_STD** | **0.07321749** | **0.00631689** | **0.06813368** | **0.11905716** | **0.05151123** |
| Sp.C.W_s1 | 1.34398736 | 0.00649621 | 0.3329238 | 1.70594884 | 1.55967033 |
| Sp.C.W_s2 | 0.39013316 | 0.03513389 | 0.60720232 | 1.30597809 | 1.10816478 |
| Sp.C.W_s3 | 0.48164409 | 0.54973246 | 0.24235522 | 1.93730335 | 1.53624028 |
| **Sp.C.W_AVG** | **0.7385882** | **0.19712086** | **0.39416045** | **1.64974343** | **1.40135847** |
| **Sp.C.W_STD** | **0.42970895** | **0.24960801** | **0.15511458** | **0.26078367** | **0.20753979** |
| Sp.O.E_s1 | 0.71594737 | 0.00065708 | 0.0217106 | 0.29801008 | 0.35454995 |
| Sp.O.E_s2 | 0.53892142 | 0.00010397 | 0.02707693 | 0.20568473 | 0.30696792 |
| Sp.O.E_s3 | 0.55240455 | 0.00027474 | 0.04271674 | 0.20193729 | 0.39264479 |
| **Sp.O.E_AVG** | **0.60242445** | **0.00034526** | **0.03050142** | **0.2352107** | **0.35138755** |
| **Sp.O.E_STD** | **0.08046133** | **0.00023125** | **0.00891104** | **0.04443221** | **0.03504884** |
| St.O.E_s1 | 0.41233197 | 0.02101314 | 0.13390466 | 0.46342671 | 0.74554554 |
| St.O.E_s2 | 0.3428498 | 0.00287241 | 0.13877801 | 0.60179949 | 0.60865121 |
| St.O.E_s3 | 0.63013664 | 0.42452982 | 0.13193791 | 0.53249827 | 0.6131093 |
| **St.O.E_AVG** | **0.4617728** | **0.14947179** | **0.13487352** | **0.53257482** | **0.65576868** |
| **St.O.E_STD** | **0.1223839** | **0.19463635** | **0.00287527** | **0.05649048** | **0.06350791** |
